# Effect of Trehalose and Sucrose in Post-thaw Quality of *Crassostrea angulata* Sperm

**DOI:** 10.3389/fphys.2021.749735

**Published:** 2021-11-25

**Authors:** Catarina Anjos, Ana Luísa Santos, Daniel Duarte, Domitília Matias, Elsa Cabrita

**Affiliations:** ^1^Centre of Marine Sciences-CCMAR, University of Algarve, Faro, Portugal; ^2^Portuguese Institute for Sea and Atmosphere-IPMA, Olhão, Portugal

**Keywords:** cryopreservation, oyster, sperm, trehalose, sucrose, cryodamage

## Abstract

Sperm cryopreservation can be a helpful tool in reproductive management and preservation of biodiversity. However, the freezing methodologies lead to some damage in structure and function of cells that may compromise post-thaw sperm activity. Cryoprotectant supplementation with sugars proved to be a successful strategy to reduce cryodamage in sperm of several species, once allowing to stabilize the plasma membrane constituents. Therefore, this study intends to understand the effects of sugars in the plasma membrane, DNA integrity, and oxidative response during Portuguese oyster sperm cryopreservation. Three cryoprotectants solutions with an initial concentration of 20% dimethyl sulfoxide (DMSO) and 20% DMSO complemented with 0.9 M trehalose or sucrose in artificial seawater were employed. Sperm samples of mature males were individually collected and diluted 1:10 (v/v) in artificial seawater followed by addition of cryoprotectants [1:1 (v/v)]. Thereafter, sperm was loaded into 0.5 ml straws, maintained at 4°C for 10 min, frozen in a programmable biofreezer at −6°C/min from 0 to −70°C, and stored in liquid nitrogen. Samples were thawed in a 37°C bath for 10 s. Several techniques were performed to evaluate post-thaw quality. Sperm motility and DNA integrity were analyzed by using computer-assisted sperm analysis (CASA) software and comet assay. Flow cytometry was employed to determine membrane and acrosome integrity and to detect intracellular reactive oxygen species (ROS) and apoptosis activity. Lipid peroxidation was determined by malondialdehyde (MDA) detection by using spectrophotometry. Sperm antioxidant capacity was evaluated through glutathione peroxidase, glutathione reductase, and superoxide dismutase. Motility was not affected by the extenders containing sugars; these compounds did not reduce the DNA damage. However, both the trehalose and sucrose protected plasma membrane of cells by increasing cell viability and significantly reducing MDA content. The same finding was observed for the ROS, where live cells registered significantly lower levels of ROS in samples cryopreserved with sugars. The activity of antioxidant enzymes was higher in treatments supplemented with sugars, although not significant. In conclusion, the addition of sugars seems to play an important role in protecting the *Crassostrea angulata* sperm membrane during cryopreservation, showing potential to improve the post-thaw sperm quality and protect the cells from cryoinjuries.

## Introduction

Bivalves represent an important aquaculture supply, which has led to a great demand for this resource and consequently an increase in its production (Wijsman et al., 2019). Therefore, aquaculture needs to diversify the species produced for a greater offer. Portuguese oyster [*Crassostrea angulata* (*C. angulata*)] is a species with high potential for aquaculture production and market acceptance. It was a key species for the European shellfish industry in the 1970s; however, due to a disease and poor remediation and management measures, the natural populations decreased (Boudry et al., 1998). Nowadays, there are only three places with pure populations of Portuguese oyster, namely, in the Sado and Mira estuary (Fabioux et al., 2002) and the Guadalquivir River (Michinina and Rebordinos, 1997). Therefore, it is important to develop techniques to ensure the conservation and recovery of native populations of the Portuguese oyster, but also to enhance and support their aquaculture production (Anjos et al., 2017; Riesco et al., 2017a; Pogoda et al., 2019).

Cryopreservation methodologies are advantageous tools that may enhance conservation strategies and reproductive technologies. The applicability of sperm cryopreservation involves the maintenance of improved genetic lines or endangered species (Martínez-Páramo et al., 2017; Riesco et al., 2017a). In addition, it allows to extend the breeding season and synchronize spawning between males and females (Hassan et al., 2015). This procedure stores reproductive cells at low temperatures. Thus, one of the major challenges of cryopreservation is to prevent the associated cryodamage involving biological structures (plasma membrane, mitochondria, and chromatin) and functions of the cell in a molecular and biochemical point of view (Cabrita et al., 2010; Sieme et al., 2016). Spermatozoa characteristics make this type of cells very prone to suffer cryodamage, mostly due to the high content of polyunsaturated fatty acids of their membranes (Cabrita et al., 2014). Osmotic and thermal stress, intracellular ice crystallization, high level of reactive oxygen species (ROS), and imbalance in antioxidant defense system are some examples of consequences that may compromise the quality of the cryopreserved material (Amidi et al., 2016). These bring up the necessity to evaluate the lethal and sublethal cryodamage. The analysis of sperm quality in invertebrates has been performed by using several techniques such as motility, membrane integrity, morphology, DNA integrity, lipid peroxidation, detection of intracellular ROS, mitochondrial membrane potential, and acrosome integrity (Akcha et al., 2012; Le Goïc et al., 2013; Vignier et al., 2017; Gallo et al., 2018, 2020). However, in cryopreservation studies, most research in bivalves applies only motility, plasma membrane integrity, and fertilization as tools to assess post-thaw sperm quality. Only a few works apply specific techniques such as malondialdehyde (MDA) content determination, DNA fragmentation, and acrosome integrity (Smith et al., 2012b; Liu et al., 2016; Riesco et al., 2019). Therefore, it is crucial to establish quality assessment methodologies that are already widely applied to mammals (Gangwar et al., 2020; Öztürk et al., 2020) and marine vertebrates (Riesco et al., 2017b; Sandoval-Vargas et al., 2021a). This will allow to understand more specifically which organelles and sperm functions are affected by cryodamage. By identifying the type of damage, it is possible to outline the strategy to prevent it. One common and successful strategy to prevent cryodamage is the supplementation of freezing media with compounds that protect cells against freezing injuries (Elliott et al., 2017). Antioxidants, proteins, and sugars are some examples of non-permeant cryoprotectants used in supplementation (Martínez-Páramo et al., 2012; Riesco et al., 2017c; Diogo et al., 2019). Sugars are large molecules that act outside of the cells, interacting with the plasma membrane. Due to their high viscosity, they interfere with the physical and chemical properties of the extender solution (Fuller, 2004). Furthermore, these are natural strategies that organisms and plants adopt to survive to adverse conditions (desiccation and freezing) (Lencioni et al., 2015; Gertrudes et al., 2017). Trehalose and sucrose are two disaccharides that have a positive effect during sperm cryopreservation when used as single or combined cryoprotectants in several species such as ram (Öztürk et al., 2020), stallions (Pérez-Marín et al., 2018), boar (Pezo et al., 2020), stone flounder (Lee et al., 2021), Greenshell™ mussel (Smith et al., 2012a), and Australian flat oyster (Hassan et al., 2017a,b). A previous work developed by our group established several steps for cryopreservation protocol of *C. angulata* sperm (type of extender, type and concentration of permeant cryoprotectant, type and sperm concentration in package and freezing rate) (Riesco et al., 2017a). Nevertheless, supplementation of permeant cryoprotectant has not been yet evaluated for *C. angulata*.

This study aims to explore the effect of trehalose and sucrose, when combined with one permeant cryoprotectant as dimethyl sulfoxide (DMSO), on the structure and functions of cryopreserved spermatozoa. Simultaneously, the different cryodamage levels that compromise the quality of the spermatozoa during cryopreservation were evaluated through motility, DNA and plasma membrane integrity, content of ROS, apoptosis activity, acrosome integrity, lipid peroxidation, and antioxidant activity.

## Materials and Methods

### Reagents

All the reagents used were acquired from Sigma-Aldrich (Saint Louis, MO, United States), unless otherwise indicated.

### Sperm Collection

**FIGURE 1:**
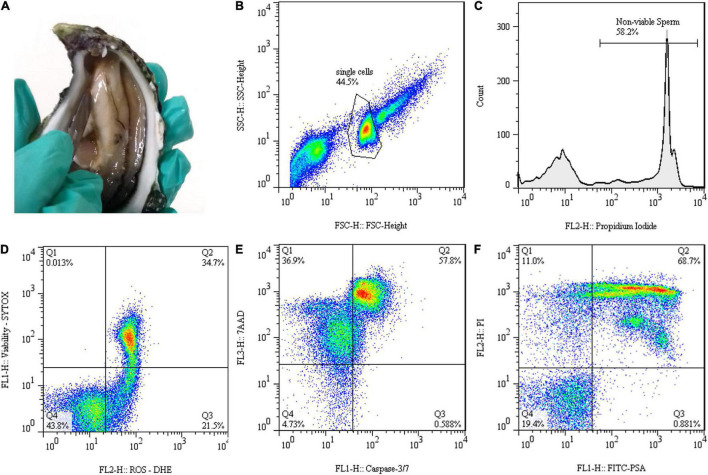
Gamete collection and flow cytometry data representation of *Crassostrea angulata* (*C. angulata*) thawed sperm: **(A)** Gonadal incisions to collect sperm. **(B)** Forward scatter (FSC-H) and side scatter (SSC-H) dot plot used to identify sperm population. **(C)** Histogram displaying propidium iodide (PI) fluorescence signal for identifying population with injured membrane. **(D–F)** Dot plots representing techniques of dual staining to detect **(D)** reactive oxygen species (ROS) by using the fluorescent probes dihydroethidium (DHE) and SYTOX^®^ Green Nucleic Acid Stain (SYTOX) [dead cells with undetectable levels of ROS (Q1); dead cells with detectable levels of ROS (Q2); live cells with detectable levels of ROS (Q3); live cells with undetectable levels of ROS (Q4)], **(E)** caspase activity applying caspase-3/7 reagent with 7-aminoactinomycin D (7-AAD) [necrotic (Q1), late apoptotic (Q2), early apoptotic (Q3) and live (Q4) cells] and **(F)** acrosome integrity by fluorescein isothiocyanate-labeled *Pisum sativum* agglutinin (FITC-PSA) and PI labeling [dead cells, acrosome intact (Q1); dead cells, acrosome reacted (Q2); live cells, acrosome reacted (Q3); live cells, acrosome intact (Q4)].

Breeders of *C. angulata* originated from one of the few pure banks of this species were acquired from the bivalve farm Viveiros Rio Mira, Lda (37°37′32.1″N, 8°41′31.6″W). Sperm was individually collected from each oyster through gonadal incisions as described by Riesco et al. (2017a) (Figure 1A). Sperm was then diluted in artificial seawater in a proportion of 1:10 and filtered with two mesh sizes (20 and 100 μM). Motility and concentration of fresh sperm were evaluated by using a computer-assisted sperm analysis (CASA) system to discard unsuitable samples. Only males with more than 40% of total motility and final concentration values between 1 and 2 × 10^9^ spermatozoa/ml were selected for freezing and thawing steps. A total of 10 sperm samples (*n* = 10) were collected and used in the following procedures.

### Cryopreservation Procedures

Sperm samples were cryopreserved with three treatments that differ in the composition of the cryopreservation solutions. Thus, cryoprotectant solutions were composed by 20% (v/v) DMSO that was defined as control or 20% (v/v) DMSO supplemented with 0.9 M (w/v) trehalose or sucrose (DMSO + Trehalose or DMSO + Sucrose, respectively). The concentration of sugars was established according to Adams et al. (2004) and Hassan et al. (2017a) for sperm cryopreservation of *Crassostrea gigas* and *Ostrea angasi*, respectively. All the solutions were prepared freshly in artificial seawater and maintained at 4°C. Each sample was mixed by using one part of prediluted sperm with one part of each cryoprotectant solution (1:1 with a final concentration of 10% DMSO and 0.45 M trehalose or sucrose) followed by equilibration of 10 min. During this time, 0.5 ml French straws were filled with diluted sperm. The straws were subjected to a freezing rate of −6°C/min from 0 to −70°C in a programmable biofreezer (Asymptote EF600, Grant Instruments Ltd., Cambridge, United Kingdom), being then immersed into liquid nitrogen to be stored in a cryobank until further analyses. Sperm thawing was performed in a bath at 37°C for 10 s, immediately before the sperm quality assessment.

### Post-thaw Sperm Quality Assessment

Sperm function or status was determined by conventional techniques such as motility and viability. However, more specific techniques (DNA fragmentation, detection of ROS and apoptosis, acrosome integrity, lipid peroxidation, and antioxidative defense) were determined to understand the extent of cryodamage and how cryoprotectant solutions applied in this trial (DMSO and DMSO supplemented with trehalose and sucrose) protect the sperm during freezing/thawing steps.

#### Motility

Computer-assisted sperm analysis system composed by ISAS software (ISAS, Proiser R + D S.L., Paterna, Valencia, Spain) was used to evaluate total sperm motility after thawing. For this, 10 μl of sample were loaded in a Makler chamber and movement was recorded with a video camera (ISAS 782C, Proiser R + D, S.L., Paterna, Valencia, Spain) connected to a phase-contrast microscope (Nikon Eclipse 200) with a 10X negative contrast objective. Software settings applied in this study were previously established by Riesco et al. (2017a) for *C. angulata* sperm, but connectivity was adjusted to 14. Motility determination was performed three times for each sample, being evaluated 10 sperm samples for each treatment (*n* = 10).

#### Deoxyribonucleic Acid Fragmentation

Comet assay was applied to quantify sperm DNA damage after thawing. This technique was performed by applying the method described for *C. angulata* sperm by Riesco et al. (2019). Spermatozoa were embedded in low melting point agarose (0.5% w/v) on agarose precoated slides. Slides were immersed in lysis solution (2.5 M sodium chloride (NaCl), 100 mM ethylenediaminetetraacetic acid disodium salt dihydrate, 10 mM Tris, 1% Triton X-100, 1% lauryl sarcosine, pH 10, 1 h at 4°C). Then, slides were immersed twice in neutralizing solution (0.4 M Tris, pH 7.5, 5 min at 4°C) and fixed in 100% ethanol (3 min). Slides were stained with propidium iodide (PI) (0.1 mg/ml) and photographed with a digital camera (F-view, Olympus Corporation, Tokyo, Japan) coupled to a fluorescent microscope (excitation filter 450–480 nm; Olympus IX 81, Olympus Corporation, Tokyo, Japan). Komet 6.0 software (Andor Technology Ltd., Belfast, United Kingdom) was used to quantify the percentage of DNA in tail of 100 cells per slide. Two slides were performed per sample (*n* = 5).

#### Flow Cytometry Approach: Membrane Integrity, Intracellular Reactive Oxygen Species Levels, Caspase Detection, and Acrosome Integrity

Flow cytometry was used to characterize the quality of post-thawed sperm. Sperm quality was evaluated through plasma membrane integrity, ROS levels, apoptosis, and acrosome integrity. Samples were acquired in the FACSCalibur Flow Cytometer (Becton Dickinson Biosciences, San Jose, CA, United States) equipped with two laser excitation sources (488 and 633 nm), two scatter detectors [forward scatter (FSC) and side scatter (SSC)], four emission detectors (FL1—530/30 nm; FL2—585/42 nm; FL3—670LP nm; FL4—661/16 nm) and controlled by CellQuest Pro version 8.7 software. For each sample, 10,000–30,000 events were registered and all the data were displayed in logarithmic mode. FSC and SSC plot were used to gate sperm population and exclude debris and aggregates from analyses (Figure 1B). Negative (unstained) and positive (single-stained) controls were used when appropriate to set gates and regions of interest and to determine compensations in the double stain protocols. FlowJo version 7.6.1 software (FlowJo, Ashland, OR, United States) was used to analyze and exhibit the flow cytometry data. Several fluorescent probes and labeling techniques were applied to evaluate different aspects of sperm physiology related to their functions or organelles.

Plasma membrane integrity of post-thaw sperm was stained by using PI. This fluorescent probe has a high affinity for DNA and is membrane impermeant, only staining sperm with the compromised plasma membrane (non-viable). Sperm with injured membrane emitted red fluorescence, which was detected at FL2 channel. The PI stock solution contained 1 mg/ml. For staining, 2.5 μl of PI was added to 10 μl of sperm and 500 μl of 1% (w/v) NaCl and incubated for 5 min in the dark. Sperm viability was quantified as the percentage of PI negative cells (membrane intact) for each sample of each treatment (*n* = 10; Figure 1C).

Reactive oxygen species levels in cryopreserved sperm were detected by using double staining with dihydroethidium (DHE, Thermo Fisher Scientific, Oregon, United States) and SYTOXR Green Nucleic Acid Stain (SYTOX, Thermo Fisher Scientific, Oregon, United States). DHE is oxidized in the presence of superoxide ions, intercalates with DNA of cells, and emits an orange fluorescence, which was captured in FL2 channel. SYTOX is a cell-impermeable DNA-binding green dye used to detect dead cells through FL1 channel. The DHE and SYTOX working solutions contained 500 and 1 μM, respectively. For staining, 5 μl of each sperm sample was diluted in 500 μl 1% NaCl and incubated in the dark with 1 μl DHE and 0.5 μl SYTOX for 10 and 5 min, respectively. The combination of dyes allowed to identify for each sample the percentage of four sperm subpopulations: dead cells with undetectable levels of ROS (Q1: DHE negative and SYTOX positive); dead cells with detectable levels of ROS (Q2: DHE and SYTOX positive); live cells with detectable levels of ROS (Q3: DHE positive and SYTOX negative); and live cells with undetectable levels of ROS (Q4: DHE and SYTOX negative) (Figure 1D). Only live sperm subpopulations were represented for each sample (*n* = 5).

Programmed cell death or apoptosis was characterized with the commercial Muse^®^ Caspase-3/7 Kit (Luminex Corporation, Austin, TX, United States). The caspase-3/7 probe is a green dye that binds to the DNA of cells in the presence of active effector caspases, producing fluorescence captured by FL1 channel. 7-aminoactinomycin D (7-AAD) fluorogenic probe also supplied in the kit, provided information that allowed differentiating between early- and late-stage apoptotic cells, by staining in red late-stage cells that were detected in the FL3 channel. The sperm labeling was carried out according to the instructions of the manufacturer. The caspase assay allowed the definition of four subpopulations of sperm: necrotic cells (Q1: caspase negative and 7-AAD positive); late apoptotic cells (Q2: caspase and 7-AAD positive); early apoptotic cells (Q3: caspase positive and 7-AAD negative); and live cells (Q4: caspase and 7-AAD negative) (Figure 1E). Data of each treatment (*n* = 8–9) were expressed as percentages for each subpopulation of sperm.

Acrosome integrity in thawed sperm was detected by combining fluorescein isothiocyanate-labeled *Pisum sativum* agglutinin (FITC-PSA) with PI. FITC-PSA fluorochrome interacts with the carbohydrate moieties and links specifically to the inner acrosomal membrane, only staining damaged acrosome. As previously stated, PI stains spermatozoa with damaged membranes. The fluorescence was measurable on FL1 and FL2 detectors for FITC-PSA and PI probes, respectively. The FITC-PSA and PI working solution contained 5 μg/ml and 1 mg/ml, respectively. For staining, 5 μl of each sperm sample was diluted in 500 μl 1% NaCl and incubated in the dark with 3 μl FITC-PSA and 2 μl PI for 10 and 5 min, respectively. Four sperm subpopulations can be identified: dead cells, acrosome intact (Q1: FITC-PSA negative and PI positive); dead cells, acrosome reacted (Q2: FITC-PSA and PI positive); live cells, acrosome reacted (Q3: FITC-PSA positive and PI negative); and live cells, acrosome intact (Q4: FITC-PSA and PI negative) (Figure 1F). Data of each treatment (*n* = 8–9) were expressed as percentages for each live subpopulation of sperm.

#### Lipid Peroxidation and Antioxidant Enzymes Activity

Spectrophotometric methods were performed for assaying lipid peroxidation and antioxidant activity in post-thaw sperm. Lipid peroxidation was determined by MDA detection by using the Bioxytech MDA-586 Kit (Oxis Research, Portland, OR, United States). Sperm samples (350 × 10^6^ cells/ml) were used to obtain cell suspensions, following the protocol developed, for fish sperm by Martínez-Páramo et al. (2012) and adapted by Riesco et al. (2019) for Portuguese oyster. According to the instructions of the manufacturer, reagents provided by the kit were added to 100 μl of each supernatant. This was read in a microplate reader at 586 nm (Synergy 4, BioTek, Vermont, United States) by using a MDA standard, provided in the kit. Each sample (*n* = 8 for each treatment) was performed in triplicate and MDA levels were expressed as nmoles of MDA per million of spermatozoa (nmol/10^6^ spz). Sperm antioxidant capacity was evaluated through superoxide dismutase (SOD), glutathione reductase (GR), and glutathione peroxidase (GPx). Selected methodologies were previously adopted by Martínez-Páramo et al. (2012) for seabass sperm. Oyster sperm samples (400 × 10^6^ cells/ml) of each treatment were centrifuged (5,000 *g*, 5 min, 4°C) to obtain the pellets. Phosphate-buffered saline (PBS) (0.01 M) with 0.1% (v/v) Triton X-100 was added to sperm pellets and submerged in liquid nitrogen (20 s) to lyse the cells, then resuspend in PBS, and centrifuge. The supernatant was split in four subsamples to determine the enzymatic activity and protein content of each sample in triplicate. The enzymatic activities of SOD, GR, and GPx were evaluated with the SOD (Ransod), GR, and GPx (Ransel) assay kits (Randox Laboratories Ltd., Crumlin, United Kingdom) according to the protocols of the manufacturer. Xanthine oxidase was the method employed by the kit to determine SOD activity, while GR activity was determined through the oxidation of NADPH and GPx activity was evaluated by NADPH oxidation in the presence of cumene hydroperoxide. Protein quantification was assessed with the Bio-Rad DC Protein Assay Kit (Bio-Rad Laboratories, United States) following producer instructions. Absorbance was determined in a microplate reader (Synergy 4, BioTek, Vermont, United States) at 505 nm for SOD, 340 nm for GR and GPx, and 750 nm for proteins. Enzymatic activity was expressed as units of enzyme per g of protein (U/g protein) for each sperm sample of each treatment (*n* = 9–10).

### Statistical Analysis

Percentage data were arcsine transformed to obtain homogenous variances (Zar, 2009). The total motility, DNA fragmentation, plasma membrane integrity, ROS levels, lipid peroxidation, and antioxidant enzyme activity parameters were analyzed through the one-way ANOVA followed by the Student–Newman–Keuls (SNK) test used to identify significant differences between cryoprotectant solutions (DMSO, DMSO + Trehalose, and DMSO + Sucrose). The results were assumed as significant at 5% level (*p* ≤ 0.05) and reported as mean ± SD. Statistical analysis was undertaken by using the software program IBM software program IBM SPSS Statistics version 25 (IBM, New York, NY, United States).

## Results

### Motility

**FIGURE 2:**
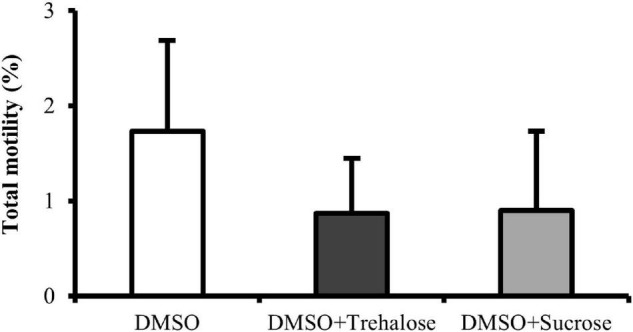
Motility of *C. angulata* sperm frozen with 10% dimethyl sulfoxide (DMSO) (white bar) and 10% DMSO supplemented with 0.45 M trehalose (black bar) or sucrose (gray bar). Results were expressed in mean percentages ± SD (*n* = 10). No significant differences between cryoprotectant solutions [one-way ANOVA followed by Student–Newman–Keuls (SNK) as *post hoc* test; *p* < 0.05].

Total motility of *C. angulata* spermatozoa after thawing was very similar for all the cryoprotectant solutions (Figure 2), showing no significant differences between treatments (DMSO: 1.73 ± 0.95%; DMSO + Trehalose: 0.87 ± 0.58%; DMSO + Sucrose: 0.90 ± 0.83%).

### Deoxyribonucleic Acid Fragmentation

**FIGURE 3:**
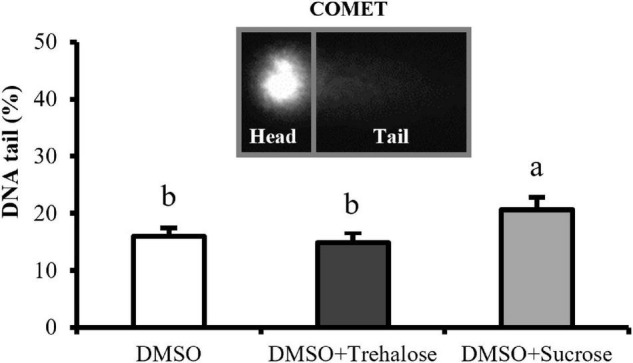
DNA fragmentation of *C. angulata* sperm frozen with 10% DMSO (white bar) and 10% DMSO supplemented with 0.45 M trehalose (black bar) or sucrose (gray bar). Results were expressed in mean percentages of DNA in the tail ± SD (*n* = 5). Different letters show differences between cryoprotectant solutions (one-way ANOVA followed by SNK as *post hoc* test; *p* < 0.05).

Deoxyribonucleic acid fragmentation results are shown in Figure 3. DMSO supplemented with sucrose had significantly higher values (20.64 ± 2.18%) of sperm with DNA damage when compared with the other two treatments. On the other hand, no significant differences were detected between DMSO supplemented with trehalose (14.95 ± 1.65%) and DMSO treatment (15.93 ± 1.47%). DNA fragmentation in thawed sperm did not seem to be reduced by sugars supplementation.

### Flow Cytometry Approach: Membrane Integrity, Intracellular Reactive Oxygen Species Levels, Caspase Detection, and Acrosome Integrity

**FIGURE 4:**
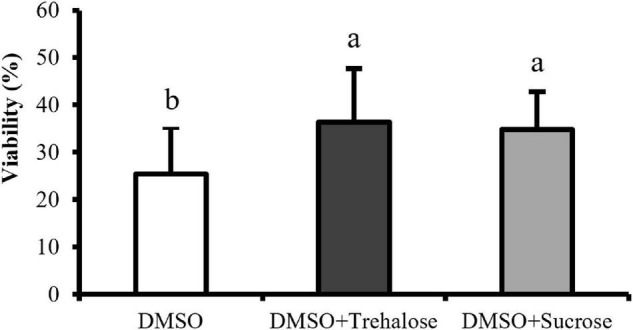
Plasma membrane integrity of *C. angulata* sperm frozen with 10% DMSO (white bar) and 10% DMSO supplemented with 0.45 M trehalose (black bar) or sucrose (gray bar) evaluated by flow cytometry. Results were expressed in mean percentages ± SD (*n* = 10). Different letters show differences between cryoprotectant solutions (one-way ANOVA followed by SNK as *post hoc* test; *p* < 0.05).

The percentage of viable cells was significantly lower in DMSO treatment (25.44 ± 9.89%) than in DMSO supplemented with sugars (Figure 4). However, no significant differences were found between trehalose (36.32 ± 11.33%) and sucrose (34.80 ± 8.04%). Therefore, the addition of both the sugars to DMSO seemed to improve post-thaw cell plasma membrane integrity, regardless of the sugar type.

**FIGURE 5:**
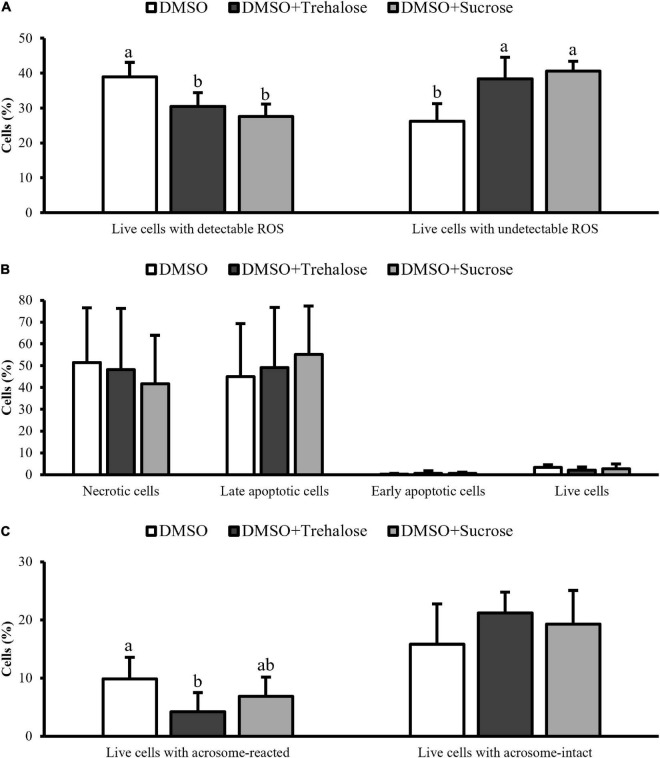
Flow cytometry assays of *C. angulata* sperm frozen with 10% DMSO (white bar) or 10% DMSO supplemented with 0.45 M trehalose (black bar) or sucrose (gray bar). **(A)** ROS levels through double staining with DHE and SYTOX. **(B)** Programmed cell death by caspase-3/7 detection. **(C)** Acrosome integrity by FITC-PSA combined with PI. Results were expressed in mean percentages ± SD (ROS: *n* = 5; caspase and acrosome: *n* = 8 for DMSO and *n* = 9 for DMSO with trehalose or sucrose). Different letters show differences between cryoprotectant solutions for each sperm subpopulation (one-way ANOVA followed by SNK as *post hoc* test; *p* < 0.05).

The levels of ROS in thawed sperm of *C. angulata* were detected with DHE and SYTOX fluorochromes being only represented the most relevant subpopulations: live sperm with detectable and undetectable levels of superoxide ions (Figure 5A). The cryoprotectants complemented with sugars showed a significantly higher percentage of living cells with undetectable levels of ROS (DMSO + Trehalose: 38.38 ± 6.21%; DMSO + Sucrose: 40.62 ± 2.73%) in comparison with DMSO (26.22 ± 5.00%). Therefore, the percentage of living cells with detectable levels of ROS was significantly higher in DMSO treatment (38.96 ± 4.18%) when compared to solutions containing sugars (DMSO + Trehalose: 30.50 ± 3.92%; DMSO + Sucrose: 27.56 ± 3.55%).

Programmed death cell (Figure 5B) allowed to identify four subpopulations: necrotic, late apoptotic, early apoptotic, and live sperm for each cryoprotectant solution. Early apoptotic and live cells were very similar between cryoprotectant solutions. DMSO showed a high percentage of necrotic cells (DMSO: 51.38 ± 25.24%; DMSO + Trehalose: 48.21 ± 28.04%; DMSO + Sucrose: 41.69 ± 22.25%), while sugar supplementation, especially sucrose, had high values of late apoptotic cells (DMSO: 44.96 ± 24.38%; DMSO + Trehalose: 49.10 ± 27.66%; DMSO + Sucrose: 55.13 ± 22.28%). However, no significant differences were found for each sperm subpopulation between treatments.

Figure 5C displays the live cells subpopulations identified in the acrosome integrity analyses. Sperm supplemented with sugars had higher values of live cells with acrosome intact (DMSO + Trehalose: 21.19 ± 3.60%; DMSO + Sucrose: 19.28 ± 5.78%) than DMSO treatment (15.85 ± 6.93%), however, with no significant differences. On the other hand, DMSO had a significantly higher number of cells with reacted acrosome (9.85 ± 3.71%) when compared to trehalose treatment (4.18 ± 3.32%).

### Lipid Peroxidation and Antioxidant Enzymes Activity

**FIGURE 6:**
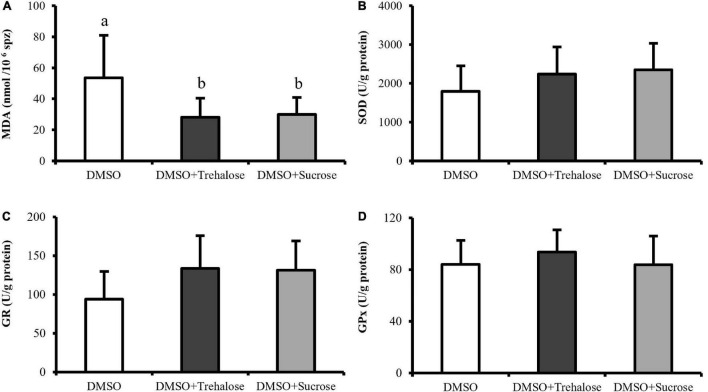
Spectrophotometric assays of *C. angulata* sperm frozen with 10% DMSO (white bar) and 10% DMSO supplemented with 0.45 M trehalose (black bar) or sucrose (gray bar). **(A)** Lipid peroxidation through malondialdehyde (MDA) levels (*n* = 8). **(B–D)** Enzyme activity of **(B)** superoxide dismutase (SOD) (*n* = 10), **(C)** glutathione reductase (GR) (*n* = 9) and **(D)** glutathione peroxidase (GPx) (*n* = 10). Results were expressed in mean ± SD. Different letters show differences between cryoprotectant solutions (one-way ANOVA followed by SNK as *post hoc* test; *p* < 0.05).

Lipid peroxidation and antioxidant enzymes are shown in Figure 6. Lipid peroxidation assay revealed that sperm frozen with DMSO exhibited significantly higher MDA contents (53.49 ± 27.41 nmol/10^6^ spz) when compared with sugar treatments (DMSO + Trehalose: 28.13 ± 12.35 nmol/10^6^ spz; DMSO + Sucrose: 29.93 ± 10.95 nmol/10^6^ spz) (Figure 6A). With respect to the activity of antioxidant enzymes, although treatments supplemented with sugars showed a trend of high enzymatic activity (Figures 6B–D), there were no significant differences between treatments.

## Discussion

Oysters are low trophic filter feeders that constitute an important resource for fisheries and aquaculture. However, due to bad management of seed production and anthropogenic impacts, some species such as the *C. angulata*, which shows potential for aquaculture diversification, are at risk of disappearing. Cryopreservation is a helpful method for genetic resources preservation. This method is a useful tool that may support the management of *C. angulata* pure natural banks and, at the same time, aquaculture production activities. However, the cryopreservation process produces cellular stress that generates damage in cells (Xin et al., 2020). Due to the complex dynamic structure of spermatozoa, it is necessary to establish different protocols for each species, toward the reduction of cryodamage. The supplementation with non-permeant cryoprotectants is a successful strategy to reduce cryodamage in cells through the use of high viscosity, high-molecular weight, and non-toxic compounds (Elliott et al., 2017). These strategies have been used in sea bass (*Dicentrarchus labrax*), dusky grouper (*Epinephelus marginatus*) and sea bream (*Sparus aurata*) improving either motility or cell viability or enhancing the antioxidant system by the addition of antioxidants (ascorbic acid, α-tocopherol), amino acids (taurine, hypotaurine), antifreeze proteins (AFPI, AFPIII), or sugars (glucose, sucrose) (Martínez-Páramo et al., 2012; Zilli et al., 2014; Riesco et al., 2017c).

In this study, sugars incorporated into the freezing media improved sperm plasma membrane integrity and reduced ROS levels, acrosome damage (only trehalose), and lipid peroxidation. This was probably due to high-molecular weight of sugars, which promoted cellular dehydration by replacing the water in the membrane that improved permeant cryoprotectant incorporation (De Leeuw et al., 1993). At the same time, due to a high viscous environment, the surrounding of the cells is stabilized, protecting the plasma membrane from cold damage (Nicolajsen and Hvidt, 1994; Woelders et al., 1997). This protective effect of sugars in post-thaw sperm was also reported for several species such as boar, Salmonidae fish, and Australian flat oyster (Gómez-Fernández et al., 2012; Nynca et al., 2016; Hassan et al., 2017a). Trehalose and sucrose have shown an important role as supplement of the freezing medium during cryopreservation of bivalves, being mainly applied in larvae (Choi et al., 2008; Labbé et al., 2018) and less in sperm (Hassan et al., 2017a; Demoy-Schneider et al., 2018). Moreover, studies in bivalve sperm were performed with a low number of quality analyses both in the fresh and post-thaw samples, being these analyses focused mainly on sperm motility, plasma membrane integrity, and fertilization success (Vitiello et al., 2011; Horváth et al., 2012; Suquet et al., 2016; Hassan et al., 2017a,b), thus lacking important information on sublethal damage. The use of a wide range of quality analyses in the establishment of a cryopreservation protocol is useful to investigate the specific structure and functions of spermatozoa that are being damaged (Cabrita et al., 2014). This information will support the establishment of strategies to mitigate cryodamage (Diogo et al., 2019; Riesco et al., 2019; Kim et al., 2020; Hossen et al., 2021). Therefore, in this study, the analysis performed on post-thaw sperm revealed the effects that trehalose and sucrose supplementation had on motility, DNA and plasma membrane integrity, ROS and apoptosis (caspases pathway) detection, acrosome integrity, lipid peroxidation, and antioxidant enzymes activities (SOD, GPx, and GR). This allowed an exhaustive evaluation of sperm quality and cell cryodamage. Sperm motility is a widely used quality indicator, since its activation is necessary for sperm to reach oocytes for successful fertilization (Cabrita et al., 2008; Boulais et al., 2019). In this study, post-thaw sperm motility did not show significant differences between cryoprotectant solutions; therefore, sugars supplementation did not have an effect on this parameter. In opposition, Hassan et al. (2017a) reported an improvement of post-thaw sperm motility of Australian flat oyster (*Ostrea angasi*) when using 10% DMSO supplemented with 0.45 M trehalose. In previous studies, our group showed that sperm motility did not seem so relevant when compared with other parameters that can jeopardize cells, due to the fact, that even with few motile cells, the long sperm motility duration of this species would allow oocytes to be fertilized (Riesco et al., 2017a). This fact is also supported in *Crassostrea virginica* by Yang et al. (2012), who showed that even low sperm motility after thawing did not compromise the ability of sperm to fertilize the oocytes and further develop into D-larvae. According to Figueroa et al. (2016), one structural damage that can compromise post-thaw sperm motility of *Salmo salar* was the damage inflicted to the mitochondria. Mitochondria is an organelle also present in bivalve sperm that participates in the synthesis of ATP, producing the majority of the energy necessary for motility (Boulais et al., 2015). For this reason, some changes or damage in this structure or metabolic pathways can compromise sperm performance. In this way, in further studies, the evaluation of mitochondrial functionality such as plasma membrane potential and ATP content should be applied, since it could help to explain the motility results.

Deoxyribonucleic acid integrity evaluation is essential, since gametes need to provide secured genetic contribution to the offspring to avoid embryo abortion (Cabrita et al., 2010). Sperm freezing with sucrose addition showed significant higher DNA damage when compared to trehalose treatment and control. In equine sperm, DNA was not affected by cryopreservation by using different trehalose and sucrose concentrations (Pérez-Marín et al., 2018). Also, El-Sheshtawy et al. (2015) tested the effect of different concentrations of trehalose and sucrose (0.05, 0.1, and 0.2 M) on post-thaw quality of bull sperm and showed that freezing media supplemented with 0.05 M trehalose and 0.05 and 0.1 M sucrose produced low levels of sperm DNA fragmentation, while higher concentrations of these sugars make the sperm more prone to DNA damage. In this study, trehalose and sucrose were only tested at 0.45 M, taking into consideration the literature in similar species (Adams et al., 2004; Hassan et al., 2017a), being necessary in further studies to evaluate the effect of other concentrations. This will allow inferring about their possible effect on DNA protection and may justify our results with sucrose treatment, as demonstrated by El-Sheshtawy et al. (2015).

The membrane stabilization promoted by trehalose and sucrose allowed an improvement of sperm plasma membrane integrity. Apart from that, the high viscosity of sugars and the reduction in ice crystal formation may have contributed to the results obtained, as demonstrated by Nicolajsen and Hvidt (1994) and Woelders et al. (1997). Hassan et al. (2017a) showed that post-thaw sperm viability of Australian flat oyster (*Ostrea angasi)* was improved when a solution with 10% DMSO complemented with 0.45 M trehalose was applied. The membrane protection through sugars supplementation also reduced lipid peroxidation. Zhu et al. (2017) showed that trehalose supplementation improved plasma membrane integrity and reduced lipid peroxidation in rabbit post-thawed sperm. Sperm plasma membrane possesses high levels of lipids, which are prone to oxidation during stress events such as cold exposure (Cabrita et al., 2014). Additionally, sugars can create bonds with the polar head of phospholipids in the plasma membrane (Anchordoguy et al., 1987), thus improving membrane protection and preventing lipid peroxidation. Altogether, these factors allowed a higher maintenance of the sperm plasma membrane integrity and stabilization during cryopreservation. Although this effect was not consistent for all the tested sugars, acrosome-reacted cells were lower in trehalose treatment when compared with control. This stabilization could be the responsible factor for acrosome protection, which is an essential structure for fertilization. It has enzymes engaged in lysing the oocyte membrane and their release allow the penetration of spermatozoa into the egg (Boulais et al., 2019). This study allowed understanding the effects of cryopreservation in different sperm structures essential to achieve successful fertilization. However, future studies are necessary to investigate fertilization success and offspring quality generated from *C. angulata* cryopreserved sperm.

Reactive oxygen species are molecules produced naturally to protect the cell and regulate signal pathways (Ighodaro and Akinloye, 2018). However, when an imbalance of ROS occurs, several cell structures containing lipids, proteins or enzymes, and chromatin may be affected by oxidative stress (Sandoval-Vargas et al., 2021b). In this study, there was a reduction in the formation of ROS in post-thaw sperm supplemented with sugars when compared to DMSO treatment. Concomitantly, low levels of lipid peroxidation were also detected in sperm treated with sugars, improving in these treatments the stabilization of the plasma membrane. Low levels of ROS and MDA associated with an improvement in rabbit sperm membrane integrity were reported by Zhu et al. (2017) when sperm was supplemented with trehalose. Trehalose and sucrose also showed a high protective effect against oxidative stress in boar post-thawed sperm by reducing the ROS levels and improving the cell viability when compared with lactose (Pezo et al., 2020). The relationship between ROS levels and lipid peroxidation was also reported by Sandoval-Vargas et al. (2021a) for coho salmon (*Oncorhynchus kisutch*) sperm, which demonstrated that post-thawed samples with high levels of superoxide anions showed high MDA content and low levels of viable sperm. In this study, the sugars seemed to reduce the damage associated with oxidative stress by protecting the lipids present in oyster sperm plasma membrane, thus avoiding their peroxidation by maintaining a balance of ROS.

Antioxidant enzymes are the first line of defense system of the cell, that keep ROS levels under control (Ighodaro and Akinloye, 2018). High levels of superoxide ions can be metabolized by SOD enzyme, which catalyzes the dismutation of these molecules to form oxygen and hydrogen peroxide, while catalase and GPx enzymes convert the hydrogen peroxide into water and oxygen (Amidi et al., 2016). Therefore, this protective mechanism acts to suppress or prevent the production of harmful molecules. Any changes in the activity of the antioxidant enzymes, such as freezing/thawing events, make the sperm more susceptible to oxidative stress. The detection of the activity of antioxidant enzymes in this study did not show any significant differences between treatments and control, although some authors have shown higher enzymatic activity in rabbit post-thawed sperm when sugars were incorporated in the freezing media (Zhu et al., 2017). This fact may suggest that sugars are not able to protect intracellular enzymes during cryopreservation and, probably, some enzyme denaturation or enzyme cell leakage may have occurred, but their effect is similar to the control.

Caspase-3/7 triggers the beginning of cell death from which the apoptosis process cannot be reversed, leading to cell alterations such as DNA and protein degradation (Elmore, 2007). Several studies have identified apoptosis activation in cryopreserved spermatozoa leading, at latter times, to a decrease in cell viability (Riesco et al., 2017c; Diogo et al., 2018). In this study, there were no significant differences in cellular apoptosis mechanisms identified through caspase-3/7 activities between control and sugar supplemented sperm. These results suggested that supplementation of sugars in the extender did not change cell death type or pathway (early apoptosis, late apoptosis, or necrosis by other mechanisms), according to the subpopulations detected by flow cytometry. The roles of cellular apoptosis and antioxidant enzymes in this study remain unclear. In future studies, these issues should be investigated, as well as, the effect of different freezing and warming rates on antioxidant enzymes activities in *C. angulata* post-thaw sperm.

This study contributed to the optimization of *C. angulata* sperm cryopreservation. Through the obtained results, it was possible to confirm selected sugars as successful freezing media, additives to mitigate cryodamage, particularly in reducing ROS production, lipid peroxidation, and improving plasma membrane integrity. In particular, trehalose may have shown a higher protective effect during cryopreservation because its molecular structure creates more connections to phospholipid polar heads than the other tested sugar (sucrose) (Tsai et al., 2018). This effect was evident in reducing acrosome-reacted cells and in the registered levels of DNA fragmentation. This study corroborates the previous findings in several species (Hassan et al., 2017b; Zhu et al., 2017; Öztürk et al., 2020) that freezing media supplementation with trehalose is a good strategy to improve post-thaw sperm quality in *C. angulata*.

## Conclusion

In *C. angulata* sperm cryopreservation, the supplementation with trehalose and sucrose of the freezing media containing DMSO improved plasma membrane integrity and reduced the oxidative stress and lipid peroxidation caused by ROS.

Sucrose supplementation did not protect chromatin and acrosome structures of *C. angulata* post-thaw sperm, revealing damage in DNA and acrosome structures.

Sperm cryopreservation with 10% DMSO and 0.45 M trehalose showed a high protective effect in *C. angulata* post-thaw sperm quality, once improved plasma membrane and acrosome integrity, and reduced lipid peroxidation and superoxide ions levels.

The establishment of a cryopreservation protocol that evaluates several post-thaw sperm quality parameters allowed to establish a strategy to mitigate cryodamage.

## Data Availability Statement

The raw data supporting the conclusions of this article will be made available by the authors, without undue reservation.

## Author Contributions

EC designed the experimental work and acquired the funding. EC and DM supervised the experimental work. CA and AS collected the samples. CA, AS, and DD performed the laboratory analyses. CA and EC wrote the manuscript. CA, DM, and EC revised and edited the manuscript. All the authors contributed to the manuscript and approved the submitted manuscript.

## Conflict of Interest

The authors declare that the research was conducted in the absence of any commercial or financial relationships that could be construed as a potential conflict of interest.

## Publisher’s Note

All claims expressed in this article are solely those of the authors and do not necessarily represent those of their affiliated organizations, or those of the publisher, the editors and the reviewers. Any product that may be evaluated in this article, or claim that may be made by its manufacturer, is not guaranteed or endorsed by the publisher.

## References

[B1] Adams S. L. Smith J. F. Roberts R. D. Janke A. R. Kaspar H. F. Robin Tervit H. (2004). Cryopreservation of sperm of the Pacific oyster (*Crassostrea gigas*): development of a practical method for commercial spat production. *Aquaculture* 242 271–282. 10.1016/j.aquaculture.2004.08.034

[B2] Akcha F. Spagnol C. Rouxel J. (2012). Genotoxicity of diuron and glyphosate in oyster spermatozoa and embryos. *Aquat. Toxicol.* 106–107 104–113. 10.1016/j.aquatox.2011.10.018 22115909

[B3] Amidi F. Pazhohan A. Nashtaei M. S. Khodarahmian M. Nekoonam S. (2016). The role of antioxidants in sperm freezing: a review. *Cell Tissue Bank.* 17 745–756. 10.1007/s10561-016-9566-5 27342905

[B4] Anchordoguy T. J. Rudolph A. S. Carpenter J. F. Crowe J. H. (1987). Modes of interaction of cryoprotectants with membrane phospholipids during freezing. *Cryobiology* 24 324–331. 10.1016/0011-2240(87)90036-8 3621976

[B5] Anjos C. Baptista T. Joaquim S. Mendes S. Matias A. M. Moura P. (2017). Broodstock conditioning of the Portuguese oyster (*Crassostrea angulata*, Lamarck, 1819): influence of different diets. *Aquac. Res.* 48 3859–3878. 10.1111/are.13213

[B6] Boudry P. Heurtebise S. Collet B. Cornette F. Gérard A. (1998). Differentiation between populations of the Portuguese oyster, *Crassostrea angulata* (Lamark) and the Pacific oyster, *Crassostrea gigas* (Thunberg), revealed by mtDNA RFLP analysis. *J. Exp. Mar. Biol. Ecol.* 226 279–291. 10.1016/S0022-0981(97)00250-5

[B7] Boulais M. Demoy-Schneider M. Alavi S. M. H. Cosson J. (2019). Spermatozoa motility in bivalves: signaling, flagellar beating behavior, and energetics. *Theriogenology* 136 15–27. 10.1016/j.theriogenology.2019.06.025 31234053

[B8] Boulais M. Soudant P. Le Goïc N. Quéré C. Boudry P. Suquet M. (2015). Involvement of mitochondrial activity and OXPHOS in ATP synthesis during the motility phase of spermatozoa in the pacific oyster, *Crassostrea gigas*. *Biol. Reprod.* 93 1–7. 10.1095/biolreprod.115.128538 26423125

[B9] Cabrita E. Martínez-Páramo S. Gavaia P. J. Riesco M. F. Valcarce D. G. Sarasquete C. (2014). Factors enhancing fish sperm quality and emerging tools for sperm analysis. *Aquaculture* 432 389–401. 10.1016/j.aquaculture.2014.04.034 25141623

[B10] Cabrita E. Robles V. Herráez P. (2008). “Sperm quality assessment,” in *Methods in Reproductive Aquaculture: Marine and Freshwater Species*, eds Cabrita E. Robles V. Herráez P. (Boca Raton, FL: CRC Press), 93–147.

[B11] Cabrita E. Sarasquete C. Martínez-Páramo S. Robles V. Beirão J. Pérez-Cerezales S. (2010). Cryopreservation of fish sperm: applications and perspectives. *J. Appl. Ichthyol.* 26 623–635. 10.1111/j.1439-0426.2010.01556.x

[B12] Choi Y. H. Lee J. Y. Chang Y. J. (2008). The influence of developmental stages and protective additives on cryopreservation of surf clam (*Spisula sachalinensis*) larvae. *J. Environ. Biol.* 29 461–463. 19195381

[B13] De Leeuw F. E. De Leeuw A. M. Den Daas J. H. G. Colenbrander B. Verkleij A. J. (1993). Effects of various cryoprotective agents and membrane-stabilizing compounds on bull sperm membrane integrity after cooling and freezing. *Cryobiology* 30 32–44. 10.1006/cryo.1993.1005 8440128

[B14] Demoy-Schneider M. Schmitt N. Le Pennec G. Suquet M. Cosson J. (2018). Quality assessment of cryopreserved black-lip pearl oyster *Pinctada margaritifera* spermatozoa. *Aquaculture* 497 278–286. 10.1016/j.aquaculture.2018.07.067

[B15] Diogo P. Martins G. Nogueira R. Marreiros A. Gavaia P. J. Cabrita E. (2019). Cryoprotectants synergy improve zebrafish sperm cryopreservation and offspring skeletogenesis. *Cryobiology* 91 115–127. 10.1016/j.cryobiol.2019.10.001 31605703

[B16] Diogo P. Martins G. Quinzico I. Nogueira R. Gavaia P. J. Cabrita E. (2018). Electric ultrafreezer (−150 ^°^C) as an alternative for zebrafish sperm cryopreservation and storage. *Fish Physiol. Biochem.* 44 1443–1455. 10.1007/s10695-018-0500-6 29654541

[B17] El-Sheshtawy R. I. Sisy G. A. El-Nattat W. S. (2015). Effects of different concentrations of sucrose or trehalose on the post-thawing quality of cattle bull semen. *Asian Pac. J. Reprod.* 4 26–31. 10.1016/S2305-0500(14)60053-1

[B18] Elliott G. D. Wang S. Fuller B. J. (2017). Cryoprotectants: a review of the actions and applications of cryoprotective solutes that modulate cell recovery from ultra-low temperatures. *Cryobiology* 76 74–91. 10.1016/j.cryobiol.2017.04.004 28428046

[B19] Elmore S. (2007). Apoptosis: a review of programmed cell death. *Toxicol. Pathol.* 35 495–516. 10.1080/01926230701320337 17562483 PMC2117903

[B20] Fabioux C. Huvet A. Lapègue S. Heurtebise S. Boudry P. (2002). Past and present geographical distribution of populations of Portuguese (*Crassostrea angulata*) and Pacific (*C. gigas*) oysters along the European and north African Atlantic coats. *Haliotis* 31 33–44.

[B21] Figueroa E. Valdebenito I. Merino O. Ubilla A. Risopatrón J. Farias J. G. (2016). Cryopreservation of Atlantic salmon *Salmo salar* sperm: effects on sperm physiology. *J. Fish Biol.* 89 1537–1550. 10.1111/jfb.13052 27406003

[B22] Fuller B. J. (2004). Cryoprotectants: the essential antifreezes to protect life in the frozen state. *Cryo Lett.* 25 375–388. 15660165

[B23] Gallo A. Esposito M. C. Cuccaro A. Buia M. C. Tarallo A. Monfrecola V. (2020). Adult exposure to acidified seawater influences sperm physiology in *Mytilus galloprovincialis*: laboratory and in situ transplant experiments. *Environ. Pollut.* 265:115063. 10.1016/j.envpol.2020.115063 32806401

[B24] Gallo A. Manfra L. Boni R. Rotini A. Migliore L. Tosti E. (2018). Cytotoxicity and genotoxicity of CuO nanoparticles in sea urchin spermatozoa through oxidative stress. *Environ. Int.* 118 325–333. 10.1016/j.envint.2018.05.034 29960187

[B25] Gangwar C. Kharche S. D. Mishra A. K. Saraswat S. Kumar N. Sikarwar A. K. (2020). Effect of diluent sugars on capacitation status and acrosome reaction of spermatozoa in buck semen at refrigerated temperature. *Trop. Anim. Health Prod.* 52 3409–3415. 10.1007/s11250-020-02374-8 32918161

[B26] Gertrudes A. Craveiro R. Eltayari Z. Reis R. L. Paiva A. Duarte A. R. C. (2017). How do animals survive extreme temperature amplitudes? The role of natural deep eutectic solvents. *ACS Sustain. Chem. Eng.* 5 9542–9553. 10.1021/acssuschemeng.7b01707

[B27] Gómez-Fernández J. Gómez-Izquierdo E. Tomás C. Mocé E. de Mercado E. (2012). Effect of different monosaccharides and disaccharides on boar sperm quality after cryopreservation. *Anim. Reprod. Sci.* 133 109–116. 10.1016/j.anireprosci.2012.06.010 22771077

[B28] Hassan M. M. Li X. Liu Y. Qin J. G. (2017a). Sperm cryopreservation in the spermcasting Australian flat oyster *Ostrea angasi* by a programmable freezing method. *Cryobiology* 76 119–124. 10.1016/j.cryobiol.2017.03.007 28341133

[B29] Hassan M. M. Li X. Qin J. G. (2017b). Improvement of post-thaw sperm survivals using liquid nitrogen vapor in a spermcasting oyster *Ostrea angasi*. *Cryobiology* 78 1–7. 10.1016/j.cryobiol.2017.08.003 28803845

[B30] Hassan M. M. Qin J. G. Li X. (2015). Sperm cryopreservation in oysters: a review of its current status and potentials for future application in aquaculture. *Aquaculture* 438 24–32. 10.1016/j.aquaculture.2014.12.037

[B31] Horváth Á Bubalo A. Čučević A. Bartulović V. Kotrik L. Urbányi B. (2012). Cryopreservation of sperm and larvae of the European flat oyster (*Ostrea edulis*). *J. Appl. Ichthyol.* 28 948–951. 10.1111/jai.12066

[B32] Hossen S. Sukhan Z. P. Cho Y. Kho K. H. (2021). Effects of cryopreservation on gene expression and post thaw sperm quality of Pacific abalone, *Haliotis discus hannai*. *Front. Mar. Sci.* 8:652390. 10.3389/fmars.2021.652390

[B33] Ighodaro O. M. Akinloye O. A. (2018). First line defence antioxidants-superoxide dismutase (SOD), catalase (CAT) and glutathione peroxidase (GPX): their fundamental role in the entire antioxidant defence grid. *Alex. J. Med.* 54 287–293. 10.1016/j.ajme.2017.09.001

[B34] Kim S. C. Hossen S. Kho K. H. (2020). Cryopreservation of sperm from farmed Pacific abalone, *Haliotis discus hannai*. *Cryobiology* 94 49–56. 10.1016/j.cryobiol.2020.04.011 32387287

[B35] Labbé C. Haffray P. Mingant C. Quittet B. Diss B. Tervit H. R. (2018). Cryopreservation of Pacific oyster (*Crassostrea gigas*) larvae: revisiting the practical limitations and scaling up the procedure for application to hatchery. *Aquaculture* 488 227–234. 10.1016/j.aquaculture.2018.01.023

[B36] Le Goïc N. Hégaret H. Fabioux C. Miner P. Suquet M. Lambert C. (2013). Impact of the toxic dinoflagellate *Alexandrium catenella* on Pacific oyster reproductive output: application of flow cytometry assays on spermatozoa. *Aquat. Living Resour.* 26 221–228. 10.1051/alr/2013047

[B37] Lee Y. H. Park J. Y. Lee I. Y. Zidni I. Lim H. K. (2021). Effects of cryoprotective agents and treatment methods on sperm cryopreservation of stone flounder, *Kareius bicoloratus*. *Aquaculture* 531:735969. 10.1016/j.aquaculture.2020.735969

[B38] Lencioni V. Jousson O. Guella G. Bernabò P. (2015). Cold adaptive potential of chironomids overwintering in a glacial stream. *Physiol. Entomol.* 40 43–53. 10.1111/phen.12084

[B39] Liu B. Liu Y. Liu S. Xu T. Liu Q. Li X. (2016). Cryopreservation of strip spawned sperm using non-programmable freezing technique in the blue mussel *Mytilus galloprovincialis*. *Aquac. Res.* 47 3888–3898. 10.1111/are.12839

[B40] Martínez-Páramo S. Diogo P. Dinis M. T. Herráez M. P. Sarasquete C. Cabrita E. (2012). Incorporation of ascorbic acid and α-tocopherol to the extender media to enhance antioxidant system of cryopreserved sea bass sperm. *Theriogenology* 77 1129–1136. 10.1016/j.theriogenology.2011.10.017 22153272

[B41] Martínez-Páramo S. Horváth Á Labbé C. Zhang T. Robles V. Herráez P. (2017). Cryobanking of aquatic species. *Aquaculture* 472 156–177. 10.1016/j.aquaculture.2016.05.042 29276317 PMC5737826

[B42] Michinina S. Rebordinos L. (1997). Genetic differentiation in marine and estuarine natural populations of *Crassostrea angulata*. *Mar. Ecol. Prog. Ser.* 154 167–174. 10.3354/meps154167

[B43] Nicolajsen H. Hvidt A. (1994). Phase behavior of the system trehalose-NaCl-water. *Cryobiology* 31 199–205. 10.1006/cryo.1994.1024

[B44] Nynca J. Judycka S. Liszewska E. Dobosz S. Grudniewska J. Arai K. (2016). Utility of different sugar extenders for cryopreservation and post-thaw storage of sperm from *Salmonidae* species. *Aquaculture* 464 340–348. 10.1016/j.aquaculture.2016.07.014

[B45] Öztürk A. E. Bodu M. Bucak M. N. Ağır V. Özcan A. Keskin N. (2020). The synergistic effect of trehalose and low concentrations of cryoprotectants can improve post-thaw ram sperm parameters. *Cryobiology* 95 157–163. 10.1016/j.cryobiol.2020.03.008 32259524

[B46] Pérez-Marín C. C. Requena F. D. Arando A. Ortiz-Villalón S. Requena F. Agüera E. I. (2018). Effect of trehalose- and sucrose-based extenders on equine sperm quality after vitrification: preliminary results. *Cryobiology* 80 62–69. 10.1016/j.cryobiol.2017.12.002 29229561

[B47] Pezo F. Zambrano F. Uribe P. Risopatrón J. Moya C. de Andrade A. F. C. (2020). Oxidative and nitrosative stress in frozen-thawed pig spermatozoa. II: effect of the addition of saccharides to freezing medium on sperm function. *Cryobiology* 97 5–11. 10.1016/j.cryobiol.2020.10.015 33121933

[B48] Pogoda B. Brown J. Hancock B. Preston J. Pouvreau S. Kamermans P. (2019). The Native Oyster Restoration Alliance (NORA) and the Berlin oyster recommendation: bringing back a key ecosystem engineer by developing and supporting best practice in Europe. *Aquat. Living Resour.* 32:13. 10.1051/alr/2019012

[B49] Riesco M. F. Félix F. Matias D. Joaquim S. Suquet M. Cabrita E. (2019). Comparative study on cellular and molecular responses in oyster sperm revealed different susceptibilities to cryopreservation. *Aquaculture* 498 223–229. 10.1016/j.aquaculture.2018.08.049

[B50] Riesco M. F. Félix F. Matias D. Joaquim S. Suquet M. Cabrita E. (2017a). First study in cryopreserved *Crassostrea angulata* sperm. *Gen. Comp. Endocrinol.* 245 108–115. 10.1016/j.ygcen.2016.05.003 27167499

[B51] Riesco M. F. Oliveira C. Soares F. Gavaia P. J. Dinis M. T. Cabrita E. (2017b). Solea senegalensis sperm cryopreservation: new insights on sperm quality. *PLoS One* 12:e0186542. 10.1371/journal.pone.0186542 29053706 PMC5650144

[B52] Riesco M. F. Raposo C. Engrola S. Martínez-Páramo S. Mira S. Cunha M. E. (2017c). Improvement of the cryopreservation protocols for the dusky grouper, *Epinephelus marginatus*. *Aquaculture* 470 207–213. 10.1016/j.aquaculture.2016.12.027

[B53] Sandoval-Vargas L. Dumorné K. Contreras P. Farías J. G. Figueroa E. Risopatrón J. (2021a). Cryopreservation of coho salmon sperm (*Oncorhynchus kisutch*): effect on sperm function, oxidative stress and fertilizing capacity. *Aquaculture* 533:736151. 10.1016/j.aquaculture.2020.736151

[B54] Sandoval-Vargas L. Silva Jiménez M. Risopatrón González J. Villalobos E. F. Cabrita E. Valdebenito Isler I. (2021b). Oxidative stress and use of antioxidants in fish semen cryopreservation. *Rev. Aquac.* 13 365–387. 10.1111/raq.12479

[B55] Sieme H. Oldenhof H. Wolkers W. F. (2016). Mode of action of cryoprotectants for sperm preservation. *Anim. Reprod. Sci.* 169 2–5. 10.1016/j.anireprosci.2016.02.004 26936658

[B56] Smith J. F. Adams S. L. McDonald R. M. Gale S. L. McGowan L. T. Tervit H. R. (2012b). Cryopreservation of Greenshell™ mussel (*Perna canaliculus*) sperm. II. Effect of cryopreservation on fertility, motility, viability and chromatin integrity. *Aquaculture* 364–365 322–328. 10.1016/j.aquaculture.2012.08.039

[B57] Smith J. F. Adams S. L. Gale S. L. McGowan L. T. Tervit H. R. Roberts R. D. (2012a). Cryopreservation of Greenshell™ mussel (*Perna canaliculus*) sperm. I. Establishment of freezing protocol. *Aquaculture* 334–337 199–204. 10.1016/j.aquaculture.2011.12.027

[B58] Suquet M. Gourtay C. Donval A. Le Goïc N. Quere C. Malo F. (2016). The quality of great scallop (*Pecten maximus*) sperm after thawing. *Gen. Comp. Endocrinol.* 229 127–131. 10.1016/j.ygcen.2016.02.023 26944486

[B59] Tsai S. Chong G. Meng P.-J. Lin C. (2018). Sugars as supplemental cryoprotectants for marine organisms. *Rev. Aquac.* 10 703–715. 10.1111/raq.12195

[B60] Vignier J. Volety A. K. Rolton A. Le Goïc N. Chu F. L. E. Robert R. (2017). Sensitivity of eastern oyster (*Crassostrea virginica*) spermatozoa and oocytes to dispersed oil: cellular responses and impacts on fertilization and embryogenesis. *Environ. Pollut.* 225 270–282. 10.1016/j.envpol.2016.11.052 28343714

[B61] Vitiello V. Carlino P. A. Del Prete F. Langellotti A. L. Sansone G. (2011). Effects of cooling and freezing on the motility of *Ostrea edulis* (L., 1758) spermatozoa after thawing. *Cryobiology* 63 118–124. 10.1016/j.cryobiol.2011.07.004 21856295

[B62] Wijsman J. W. M. Troost K. Fang J. Roncarati A. (2019). “Global production of marine bivalves. Trends and challenges,” in *Goods and Services of Marine Bivalves*, eds Smaal A. C. Ferreira J. G. Grant J. Petersen J. K. Strand Ø (Cham: Springer International Publishing). 10.1007/978-3-319-96776-9

[B63] Woelders H. Matthijs A. Engel B. (1997). Effects of trehalose and sucrose, osmolality of the freezing medium, and cooling rate on viability and intactness of bull sperm after freezing and thawing. *Cryobiology* 35 93–105. 10.1006/cryo.1997.2028 9299101

[B64] Xin M. Niksirat H. Shaliutina-Kolešová A. Siddique M. A. M. Sterba J. Boryshpolets S. (2020). Molecular and subcellular cryoinjury of fish spermatozoa and approaches to improve cryopreservation. *Rev. Aquac.* 12 909–924. 10.1111/raq.12355

[B65] Yang H. Hu E. Cuevas-Uribe R. Supan J. Guo X. Tiersch T. R. (2012). High-throughput sperm cryopreservation of eastern oyster *Crassostrea virginica*. *Aquaculture* 344–349 223–230. 10.1016/j.aquaculture.2012.03.018

[B66] Zar J. H. (2009). *Biostatistical Analysis.* Upper Saddle River, NJ: Prentice Hall.

[B67] Zhu Z. Fan X. Pan Y. Lu Y. Zeng W. (2017). Trehalose improves rabbit sperm quality during cryopreservation. *Cryobiology* 75 45–51. 10.1016/j.cryobiol.2017.02.006 28223021

[B68] Zilli L. Beirão J. Schiavone R. Herraez M. P. Gnoni A. Vilella S. (2014). Comparative proteome analysis of cryopreserved flagella and head plasma membrane proteins from sea bream spermatozoa: effect of antifreeze proteins. *PLoS One* 9:e99992. 10.1371/journal.pone.0099992 24941006 PMC4062426

